# Enhancing D-lactic acid production from non-detoxified corn stover hydrolysate via innovative F127-IEA hydrogel-mediated immobilization of *Lactobacillus bulgaricus* T15

**DOI:** 10.3389/fmicb.2024.1492127

**Published:** 2024-12-05

**Authors:** Yuhan Zheng, Feiyang Sun, Siyi Liu, Gang Wang, Huan Chen, Yongxin Guo, Xiufeng Wang, Maia Lia Escobar Bonora, Sitong Zhang, Yanli Li, Guang Chen

**Affiliations:** ^1^College of Life Science, Jilin Agricultural University, Key Laboratory of Straw Comprehensive Utilization and Black Soil Conservation, Education Ministry of China, Changchun, Jilin, China; ^2^Northeast Institute of Geography and Agroecology, University of Chinese Academy of Sciences, Beijing, China; ^3^Key Laboratory of Wetland Ecology and Environment, State Key Laboratory of Black Soils Conservation and Utilization, Northeast Institute of Geography and Agroecology, Chinese Academy of Sciences, Changchun, China; ^4^Biotechnology Research and Development Center, Vegetable and Flower Science Research Institute of Jilin Province, Changchun, China

**Keywords:** corn stover, F127-IEA, phenolic resistance, immobilization, cell-recycle fermentation

## Abstract

**Background:**

The production of D-lactic acid (D-LA) from non-detoxified corn stover hydrolysate is hindered by substrate-mediated inhibition and low cell utilization times. In this study, we developed a novel temperature-sensitive hydrogel, F127-IEA, for efficient D-LA production using a cell-recycle batch fermentation process.

**Results:**

F127-IEA exhibited a porous structure with an average pore size of approximately 1 μm, facilitating the formation of stable *Lactobacillus bulgaricus* clusters within the gel matrix. It also maintains excellent mechanical properties. It also maintains excellent mechanical properties. F127-IEA immobilized *Lactobacillus bulgaricus* T15 (F127-IEA-T15) can be used in cell-recycle fermentation for over 150 days from glucose and 50 days from corn stover hydrolysate, achieving high production rates of D-LA from glucose (2.71 ± 0.85 g/L h) and corn stover hydrolysate (1.29 ± 0.39 g/L h). F127-IEA-T15 enhanced D-LA production by adsorbing and blocking toxic substances present in corn stover hydrolysate that are detrimental to cellular activity.

**Conclusions:**

The newly developed hydrogels in this study provide a robust platform for large-scale extraction of D-LA from non-detoxified corn stover.

## 1 Introduction

Biorefineries derived from lignocellulosic biomass play a pivotal role in the emerging bioeconomy and efforts to advance energy and environmental sustainability (Partovi et al., 2020; Wang X. et al., 2020). Extensive research has focused on the conversion of lignocellulosic materials, including agricultural residues such as corn stover (Zhang et al., 2020), maize stover (Zhang et al., 2023), sugarcane bagasse (Agrawal and Kumar, 2023) as well as forestry byproducts like spruce (Campos et al., 2023), and eucalyptus (Cabrera et al., 2024), into lactic acid, underscoring their potential for sustainable bioprocessing (Ajala et al., 2020). Among various lignocellulosic sources, corn stover with an annual output of 700 million tons stands out as a crucial resource. Corn stover as a fermentation raw material is also conducive to solve global challenges such as food crisis, low utilization rate of corn stover, and environmental pollution caused by excessive burning (Li et al., 2021; Schroedter et al., 2021). The fermentable sugars from corn stover can provide an exceptional carbon source for biochemicals production. However, the pretreatment of corn stover, a key step in generating fermentable sugars, results in the formation of inhibitors such as ferulic acid (FA) and vanillin, which significantly impede microbial fermentation (Dordevic et al., 2019; Sharma et al., 2021; Zhang et al., 2021). The antimicrobial properties of polyphenols are mainly determined by the structure of the individual, such as FA through the interaction with the bacterial cell membrane, changing the hydrophobicity of the cell membrane causing local damage to the cell membrane and leakage of essential intracellular components (Li et al., 2022). These inhibitors penetrate the cell membrane, inducing the accumulation of reactive oxygen species that alter the ratio of cell membrane proteins to lipids and cause irreversible membrane damage (Sharma et al., 2009). Although detoxification processes can reduce the phenolic content in pretreated corn stover, the concentration process of corn stover hydrolysate may increase the levels of these toxic substances (Si et al., 2018). In addition, many previous studies have confirmed that a large amount of water is consumed during the washing and detoxification process, while wastewater containing large amounts of acids, alkalis and nutrients is not suitable for direct discharge into the environment without additional treatment (Gai et al., 2015).

D-LA, a subset of lactic acid (LA), is extensively utilized in the pesticide and chemical industries (Skonberg et al., 2021). It also serves as a precursor for synthesizing new polylactic acid (PLA) materials, which are promising alternatives to traditional plastics due to their biodegradability and durability (Wu et al., 2021). Consequently, there is a high global demand for D-LA, predominantly produced through microbial fermentation, which yields D-LA with superior optical purity compared to chemical synthesis (de Albuquerque et al., 2021). Approximately 90% of D-LA production currently relies on microbial fermentation technology (John et al., 2009).

Recent research has focused on immobilization techniques to enhance LA or D-LA yields from waste or non-edible food resources, though these studies have reported relatively low yields (Bahry et al., 2019; Shahri et al., 2020; Suwannakham and Yang, 2005; Zhao Z. et al., 2016). The development of novel cell immobilization methods is essential to address the challenges of low yield and poor tolerance to toxic substances in these strains (Lappa et al., 2022; Radosavljevic et al., 2020). In this context, the novel hydrogel material poloxamer F127 has gained considerable attention due to its three-dimensional cross-linked network. This hydrophilic polymer has a three-dimensional cross-linked network and is widely used in medicine and the chemical industry (Butelmann et al., 2021; Yuan et al., 2021; Zhao Z. et al., 2016). The porous matrix of F127 adsorbs phenolic inhibitors, protecting the immobilized cells from inhibitory effects (Santana et al., 2024). Additionally, F127 has excellent temperature sensitivity, transitioning between liquid and solid states depending on the temperature. It also demonstrates photosensitivity (Padaga et al., 2024). However, the mechanical properties and adsorption capacity of F127 cannot satisfy the field of hydrolysate fermentation of undetoxified corn stover (Shamma et al., 2022). Hence, we employ imino groups to improve F127′s molecular structure, along with hydrogen bonds and π-π interactions, enhances its adsorption capacity for aromatic compounds. The cell-recycle fermentation process reduces cellular load and enhances cell density, promoting a robust cell population that improves glucose utilization and D-LA production (Gupta et al., 2024; John and Nampoothiri, 2011; Liu et al., 2022). In this study, we innovatively prepared and employed F127-IEA for immobilizing *Lactobacillus bulgaricus* T15 to produce D-LA through cell-recycle fermentation from corn stover (Supplementary Figure S2).

## 2 Materials and methods

### 2.1 Materials

For the measurement of glucose content during the fermentation process, a glucose kit was purchased from Shanghai Rongsheng Biopharmaceutical (Shanghai, China). Pluronic-F127 was obtained from Sigma-Aldrich Corp. (MO, USA); C_7_H_9_NO_3_ from Shanghai Yuan-ye (Shanghai, China), and Cellic CTec 3 (Enzyme activity: 1000 BHU-2-HS/g) from Novozymes (Copenhagen, Denmark). All other reagents were of analytical grade.

### 2.2 Strain and cultural condition

The strain used was *Lactobacillus* sp. T15, provided by the key laboratory of the Ministry of Education of stover Comprehensive Utilization and Black Soil Preservation (Jilin Province, China). The MRS medium used in this experiment was purchased from Aladdin (Shanghai, China). The ferment condition for *Lactobacillus* sp. T15 before and after immobilization were 41°C, 2% inoculation, and an initial glucose concentration of 80 g/L. Under these specified conditions, the metabolic activity of the microorganisms is markedly enhanced, leading to a substantial increase in lactic acid production (Lupu et al., 2023).

### 2.3 Synthesis of F127-IEA

With slight modifications based on the method of Li et al. (2023). The procedure began with the drying of 30 g of F127 powder at room temperature for 16 h. Subsequently, anhydrous CH_2_Cl_2_ (250 mL) was added to the reactor under a nitrogen atmosphere, and the system was maintained at 30°C. Dibutyltin laurate (six drops) and ethyl isocyanate acrylate (25 mL) were added, with the latter added at a rate of approximately one drop per second. The reaction proceeded at 30°C for 2 days. Upon completion, methanol (30 mL) was added to terminate the reaction. The reaction mixture was then transferred to a rotary evaporator and concentrated at 30°C. The concentrated solution was subsequently added to 1 L of ether and stirred for 15 min before being dispensed into 50 mL centrifuge tubes. The precipitate was obtained by centrifugation at 1,288 g for 10 min, and the supernatant was discarded. This centrifugation process was repeated twice with additional ether to ensure the removal of impurities from the precipitate (Supplementary Figure S1).

### 2.4 Preparation of F127-IEA immobilized *Lactobacillus sp*. T15 (F127-IEA-T15)

Three grams of F127-IEA were dissolved in 6 mL of sterile deionized water and stored at 4°C overnight. The solution was then heated to 28°C for gel conversion to verify its temperature sensitivity, and subsequently transferred to an ice-water mixture. The photo-initiator 2-hydroxy-2-methylpropyl phenyl ketone (20 μL/10 g hydrogel solution) was added to F127-IEA in a ratio of 1:10 with the activated T15 seed solution. Then cured at 365 nm for 1 h (Johnston et al., 2020).

### 2.5 Morphology, structure and mechanical properties analysis of F127-IEA and F127-IEA-T15

Before analysis, the desired sample of F127-IEA and F127-IEA-T15 for SEM (cryogenic scanning electronic microscopy) was placed on 10 μL of conductive carbon adhesive. The sample stage containing the droplets was then frozen in liquid nitrogen for 30 s. The frozen sample was transferred to the preparation chamber under vacuum (~-200 Pa) for carbon plating. After sublimation at −9°C for 10 min, a 5 nm thick gold layer was sputtered on the sample surface. Samples were pre-cooled (~140°C) operating at 5 kV and imaged in an FEI Quanta450. After taking frozen SEM images, samples were analyzed using the imaging viewing software image J. The specific surface area and average porosity size of F127-IEA were compared using BET to verify if the material is a porous matrix. The C, N, and H content of F127, F127-IEA, and F127-IEA/T15 samples were measured using an elemental analyzer (German, UNICUBE^®^) to verify the success of the material modification and immobilization of T15. The TA-XT Physical Properties Analyzer (USA) was applied to the uniaxial stress compression test. The parameters of the fixture were a compression drop rate of 1 mm/s and 80% compression strain. The primary parameters used for mechanical strength analysis were strain fracture and Young's modulus. As the cross-sectional area within the microcapsule varies during the test, modifications were made using the p/36R probe and compression mode according to Equations 1, 2. The specific results are converted into Hencky stress (σH) and Hencky strain (εH), respectively (Zhao X. et al., 2016).


(1) $$\begin{array}{l} \sigma \text{H}=\frac{{\text{F}}_{\left(\text{t}\right)}\cdot {\text{H}}_{\left(\text{t}\right)}}{\left({\text{H}}_{0}\cdot {\text{A}}_{0}\right)} \end{array}$$

(2) $$\begin{array}{l} \varepsilon \text{H}=-\ln\left[\frac{{\text{H}}_{\left(\text{t}\right)}}{{\text{H}}_{0}}\right]\; \end{array}$$

F(t) is the stress at time t (N); H(t) is the height of the sample at time t (mm); A0 is the initial cross-sectional area of the sample (mm^2^); H0 is the initial height of the sample (mm). The σH-ε stress and strain corresponding to the highest point of the H curve are the fracture stress and fracture strain. Young's modulus is σH-ε and represents the gradient of the linear part of the H curve at the beginning.

Riboflavin is a typical alternative for measuring the cell encapsulation rate in hydrogel. The riboflavin instead of the T15 seed solution was dissolved in water, stirred overnight at room temperature (avoiding light), co-mixed with the riboflavin solution, and freeze-dried. A portion of the powder was solubilized in distilled water, stirred overnight, and then centrifuged for 20 min at 5,152 g. The supernatant was collected, and its OD value was measured at 445 nm using a UV-vis spectrophotometer (Cary 60 UV-Vis, Agilent, USA). The encapsulation rate was calculated according to the following equation (Butelmann et al., 2021; Yuan et al., 2021):


(3) $$\begin{array}{l} \text{Embedding rate}=\frac{{\text{C}}_{0}}{{\text{C}}_{1}}\times 100\%\; \end{array}$$

Where C_0_ is the amount of riboflavin in the gel and C_1_ is the total riboflavin content.

F127-IEA hydrogels were prepared and added to 1.5 mL centrifuge tubes. After weighing, the gels were centrifuged at 14,167 g for 15 min. The remaining gel was removed after centrifugation, and the residual weight was measured. The water retention rate of different gels was calculated using the following equation (Wang et al., 2024).


(4) $$\begin{array}{l} \text{Water retention rate}=\frac{{\text{W}}_{2}-{\text{W}}_{0}}{{\text{W}}_{1}-{\text{W}}_{0}}\times 100\% \end{array}$$

Where W_0_ is the mass of the empty centrifuge tube (g), W_1_ is the mass of the centrifuge tube with gel before centrifugation (g), and W_2_ is the mass of the centrifuge tube with gel after centrifugation (g).

### 2.6 Phenolic compounds adsorption test of F127-IEA

Equal amounts of F127-IEA gels (3 mL) were added to phenolic solutions (anhydrous ethanol, deionized water, pH 6), which contained either FA dissolved in anhydrous ethanol or vanillin dissolved in deionized water at concentrations of 0, 0.5, 1, 1.5, and 2 g/L. These mixtures were left to stand at 41°C to simulate the fermentation process. Samples were taken every 20 min to measure the concentration of the corresponding substances, which were then used to calculate the removal rate of inhibitors according to Equation 5 (Guo et al., 2022).


(5) $$\begin{array}{l} R=\frac{C_{0}-C_{e}}{C_{0}m}\times 100\;\% \end{array}$$

Where *R* is the adsorption amount at equilibrium (%), *C*_0_ is the initial concentration of the inhibitor in solution (g/L), and *C*_*e*_ is the equilibrium concentration of the inhibitor in solution (g/L). After the adsorption reached equilibrium, F127-IEA was transferred to a solution of the respective solvents for 2 h (equilibrium time for the above test). Subsequently, the content of the corresponding substance in the solution was measured to verify the adsorption strength of F127-IEA.

### 2.7 Phenolic substances tolerant test of F127-IEA-T15

The tolerance of F127-IEA-T15 cells to phenolic substances was analyzed using free T15 cells as a control. Phenolic substances (FA and vanillin) were added to the medium until the ultimate concentrations of 0, 0.5, 1, 1.5, and 2 g/L were reached. Tolerance was analyzed by measuring D-LA production as an indicator through 72 h of fermentation.

### 2.8 Preparation of non-detoxified corn stover hydrolysate

The corn stover pretreatment process was carried out in a 250 mL reactor. Initially, the corn stover at a mass fraction of 8% was mixed with the pretreatment agent (NaOH/urea: 8 wt%/12 wt%) at 80°C for 20 min. The pre-treated samples were subjected to solid-liquid separation and then dried to a constant weight in an oven at 80°C. In this study, the solvent system consisted of 2.5 mL of citrate buffer (0.05 M, pH 5.0) and 34.8 mL of distilled water, to which 1.579 g of pretreated corn stover was added for the enzymatic hydrolysis reaction. The pH was adjusted to 4.6–6.0 and cooled to room temperature. Subsequently, 2.5 mL of cellulase was added, and the sample was brought to a preheated 180 rpm rotary shaker at 50°C and stirred for 72 h. After this, the samples were centrifuged at 1,278 g for 5 min, and the supernatant was collected to obtain the corn stover enzymatic hydrolysate (Wang et al., 2019).

### 2.9 Cell-recycle fermentation via F127-IEA-T15 cells from glucose and corn stover hydrolysate

F127-IEA immobilized T15 cells-recycle fermentation was performed in 12 mL shaking tubes at 41°C and 80 g/L glucose concentration. Free cell-recycle fermentation was conducted after inoculation of 6% T15/F127-IEA microcapsules into the tubes. To assess the yield, 500 μL of fermentation broth was collected from each cycle, the mass of the remaining gel beads and the glucose concentration were measured, and the yield per gram of gel beads was calculated. The residual gel column after solid-liquid separation (filtration) was transferred to the next fermentation stage in the same medium as the previous cycle. To validate the recoverability of the various materials, the microcapsules were collected after 72 h of fermentation in each cell-recycle and their residual mass was determined. The efficacy of F127-IEA as an immobilized material was verified using corn stover hydrolysate as the sole carbon source for D-LA production via F127-IEA-T15 cells. D-LA production served as the indicator of efficiency.

### 2.10 Determination of phenols by HPLC

Phenols (FA and vanillin) in corn digest were detected using a Waters 1525 instrument equipped with a PRONTOSIL 120-10-C18 H column (250 × 4.6 mm) (Bischoff, Leonburg, Germany). Detection wavelengths were set to 210 and 321 nm, respectively, with flow rates of 0.8 and 280 mL/min, respectively.

Fermentation broth samples were filtered through a 0.2 μm syringe filter (Wheaton Science, Millville, Worcester, MA, USA). D-LA was detected using a Waters 1525 detector equipped with an Astec CLC-L column (250 × 4.6 mm) (Supelco, Bellefonte, PA, USA) with a detection wavelength of 245 nm and a flow rate of 1.2 mL/min. Additionally, acetic acid, a fermentation by-product, was determined using a Waters 1525 detector with a detection wavelength of 210 nm and a flow rate of 0.8 mL/min.

## 3 Results and discussion

### 3.1 Preparation and characterization of F127-IEA hydrogel matrix before and after immobilization of *Lactobacillus bulgaricus* T15

**Figure 1:**
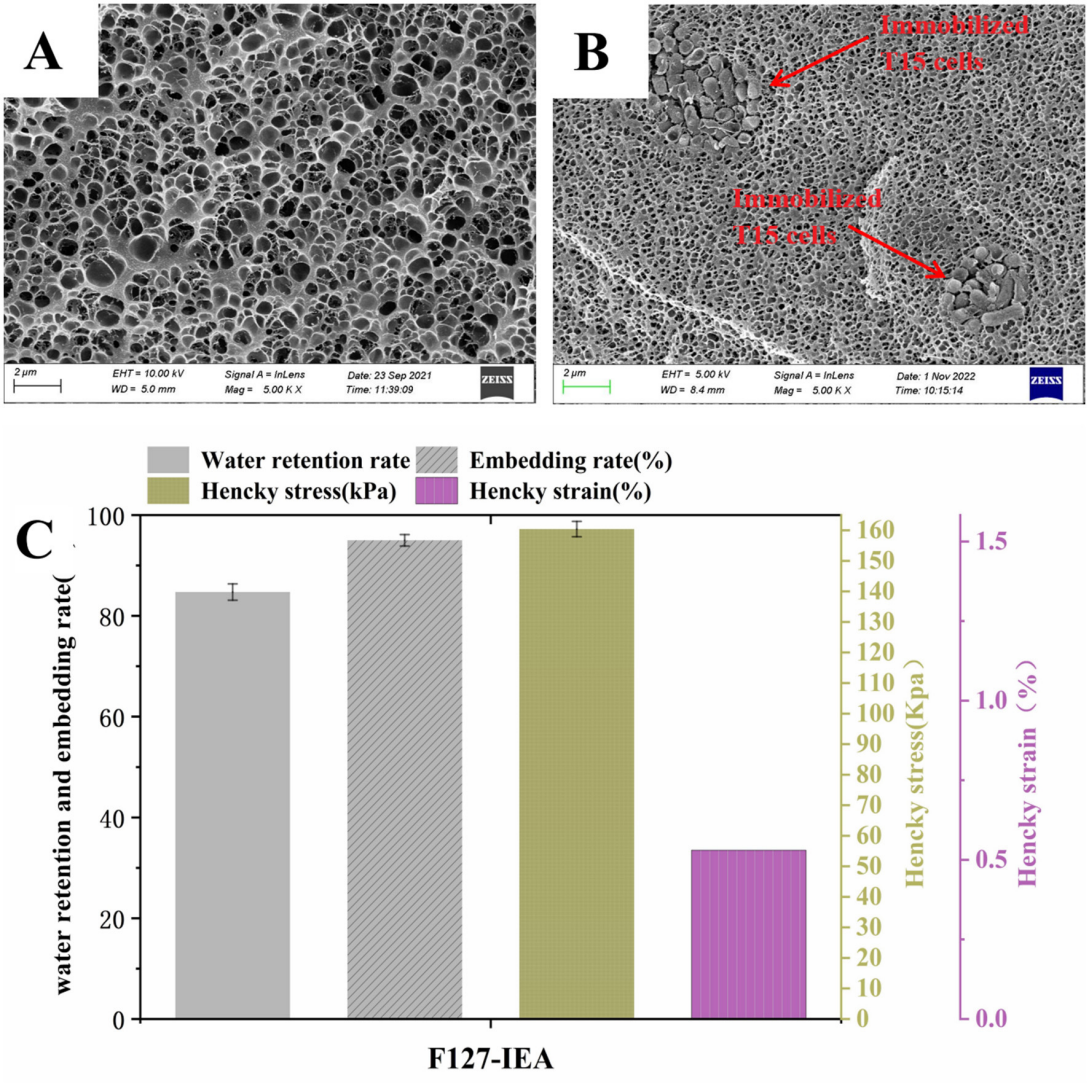
Morphology, structure and mechanical properties analysis of F127-IEA and F127-IEA-T15. SEM microstructure of F127-IEA **(A)** and F127-IEA-T15 **(B)**; the physical properties of F127-IEA **(C)**.

The thermoresponsive material, F127-IEA, was synthesized by polymerizing isocyanate ethyl ester with the imine groups at both termini of the F127 hydrogel. Subsequently, we mixed *Lactobacillus bulgaricus* T15 with F127-IEA and added a photo-initiated crosslinker (2-hydroxy-2-methylpropiophenone) to stably immobilize the T15 cells within the F127-IEA gel matrix. The nitrogen content of F127 alone was 0%, whereas the nitrogen content of F127-IEA was 0.27%, indicating successful polymerization of the C-O bonds at both ends of F127 with the carbon-oxygen double bonds of ethyl is cyanoacrylate. Following the immobilization of T15, the nitrogen content in F127-IEA increased significantly to 7.76%, while the relative contents of carbon and hydrogen decreased, confirming the successful formation of the F127-IEA gel (Supplementary Figure S1). The F127-IEA hydrogel exhibits a porous, matrix-like structure (Figure 1A), characterized by a thin-walled and uniformly distributed network with an average pore diameter of ~40.2 μm. The presence of pore channels provides a substantial surface area for bacterial cell attachment. The T15 cells, with an average diameter of ~1 μm, were immobilized within the gel's pores. The cells establish a stable population within the material, with newly formed cells circulating during cell division and apoptosis, thereby enhancing the population's stability and robustness over time (Figure 1B). The SEM image of the F127-IEA hydrogel after immobilizing *Lactobacillus bulgaricus* T15 is presented in Figure 1B, demonstrating the preserved porous network structure. The gel's pores effectively accommodate T15 cells, which have an average diameter of ~1 μm. These cells establish a stable population within the material, forming clusters that expand the hydrogel micropores through cell division and apoptosis. This dynamic process enhances the long-term stability and robustness of the population.

The mechanical properties, encapsulation rate, and water-holding capacity of F127-IEA were evaluated to assess its resistance to wear during stirred deep aeration fermentation. We compared these properties to those of calcium alginate gel beads, a material commonly used in similar studies. Our findings revealed that F127-IEA exhibits a Hencky stress of 159 kPa and a Hencky strain of 0.5% (Figure 1C). F127-IEA shows a higher encapsulation rate (94.9%) than calcium alginate gel beads (80.2%). This superior encapsulation enhances *Lactobacillus* sp. T15 retention, improving product yield in a limited fermentation substrate. This superior encapsulation capacity of F127-IEA for *Lactobacillus* sp. T15 is expected to lead to higher product yields within the confines of the fermentation substrate. The water-holding capacity of a material can significantly influence the stability of the immobilized carrier during fermentation. Water loss may result in the collapse of the internal mesh structure, adversely affecting the physiological activity of the cells embedded within the carrier. The water-holding capacity of F127-IEA is 84.7% (Figure 1C), 161.41% higher than that of calcium alginate, indicating its superior ability to retain water. Relative to other investigations in the field (Gajic et al., 2023), our study reveals that F127-IEA maintains a superior position in terms of Water-Retaining Capacity. Specifically, the outcomes of study demonstrated that the sodium alginate prepared therein exhibited a Water-Retaining Capacity of 76%, which was a good performance. Nevertheless, F127-IEA surpassed this benchmark, boasting a notably higher Water-Retaining Capacity by ~11.5%. This enhancement underscores the material's potential for superior performance in applications where water retention is a critical factor. These results indicate that F127-IEA possesses superior mechanical strength over the conventional calcium alginate. Its robust resistance to high-strength external forces is pivotal for maintaining durability during subsequent fermentation stirring processes. Collectively, these attributes suggest that F127-IEA is poised to become an exemplary material for cell immobilization in biotechnological applications.

### 3.2 D-LA production from glucose with F127-IEA-T15 via cell-recycle fermentation

**Figure 2:**
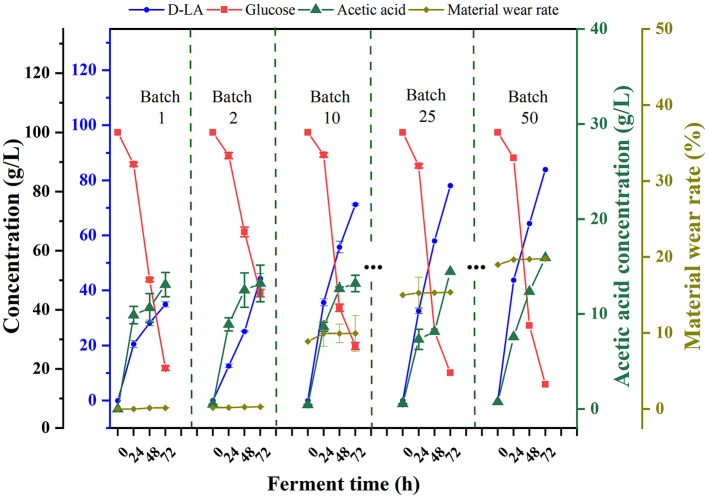
Sequential batch fermentation of glucose for production of D-LA using F127-IEA-T15 with cell recycle. Fermentation temperature was maintained at 37°C, pH at 6.5. The concentrations of glucose (red square), D-LA (blue circle), acetic acid (green triangle), material wear rate (yellow-green diamond).

To validate the applicability of F127-IEA-T15 in repeated cell-recycling fermentation, we conducted experiments producing D-LA from glucose. As shown in Figure 2, after 150 days (50 fermentation batches), the F127-IEA gel exhibited a loss rate of 15%. During the first two batches, the cells within the hydrogel had not yet reached their maximum performance and were in an adaptation phase, with D-LA production at only 40 g/L. Subsequently, the production stabilized at 80 g/L per batch, with acetic acid as a byproduct produced at 20 g/L per batch. This immobilization method increased the average yield of D-LA from glucose substrate by 30–40 g per batch. Throughout the fermentation process, the glucose consumption rate remained relatively consistent. Compared to the other studies (Qiu et al., 2023; Sansatchanon et al., 2023; Zhang et al., 2018), our study achieved an increase of 30–40 grams in the average yield per batch of D-LA production from glucose substrate through the immobilization method.

In evaluating production efficiency, our study demonstrated superior fermentation performance compared to other analogous immobilized cell fermentation studies (Wang J. et al., 2020). Specifically, we achieved a higher fermentation efficiency of 2.71 ± 0.85 g/L h over extended single fermentation cycles, totaling 72 h and 50 batches. This efficiency surpasses the 2.213 ± 0.008 g/L h observed in studies with 48-h, 8-batch cycles. Additionally, the initial glucose concentration in our study was set at a more modest 80 g/L, which is lower than the 115.3 g/L typically used in other studies. These findings underscore that F127-IEA is not only suitable for long-term fermentation production but also exhibits enhanced production efficiency at lower initial sugar concentrations. This suggests that F127-IEA could offer a more cost-effective and sustainable approach to fermentation processes, particularly when glucose resources are limited.

To simulate the equipment maintenance process, we collected the F127-IEA hydrogel after the 10th fermentation batch (30 days) and stored it at −80°C for 3 days. In subsequent fermentation experiments, we found that the freezing storage did not affect its fermentation performance, and the ability to produce D-LA after ultra-low temperature preservation demonstrated its robustness.

### 3.3 D-LA production from corn stover hydrolysate with F127-IEA-T15 via cell-recycle fermentation

**Figure 3:**
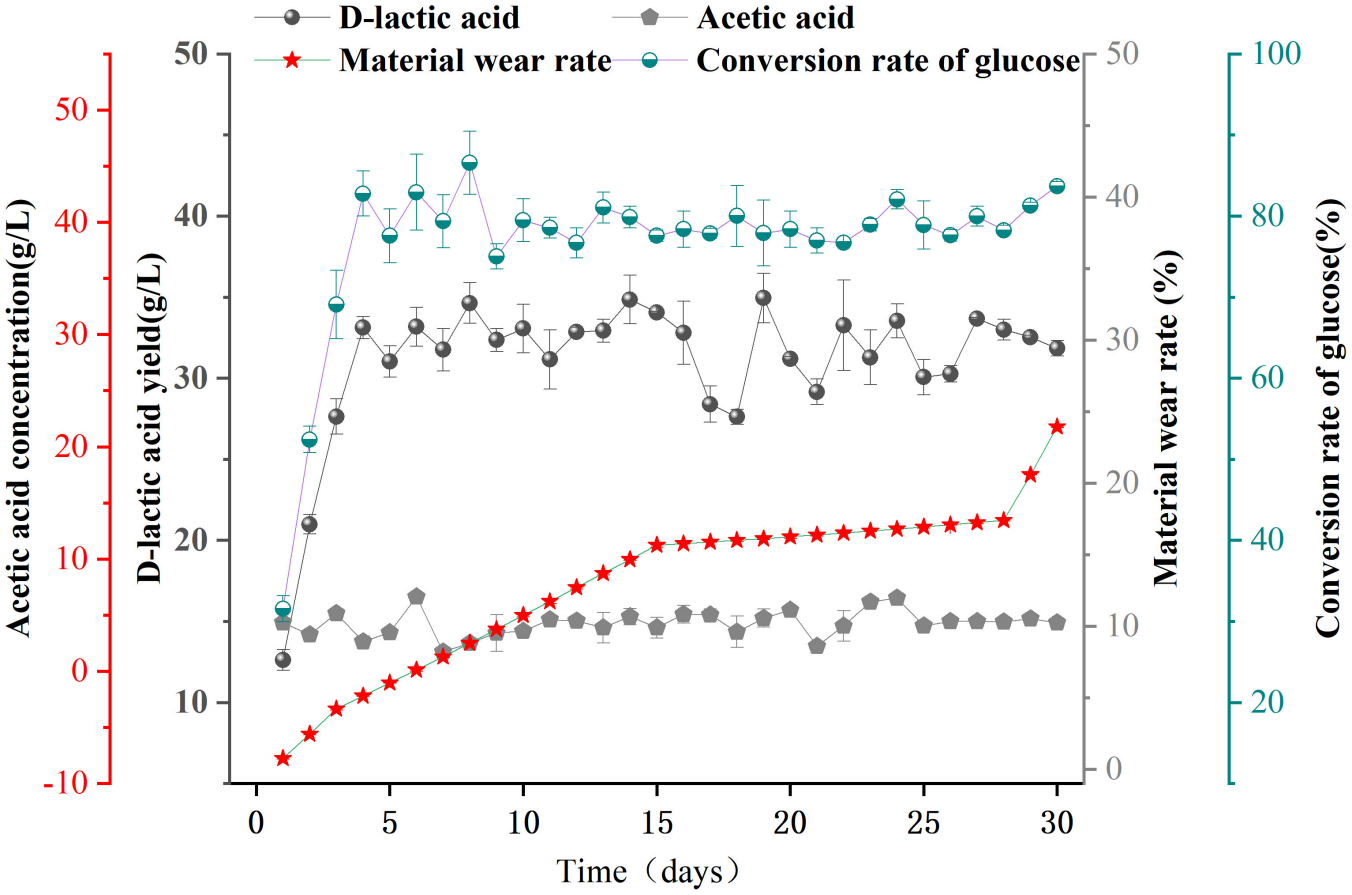
Sequential batch fermentation of non-detoxified corn stover hydrolysate for D-LA production using F127-IEA-T15 with cell recycle. Fermentation temperature was maintained at 37°C, pH at 6.5. The concentrations of D-LA (black spherical), acetic acid (gray pentagon), material wear rate (red five-pointed star), and conversion rate of glucose (half dark green, half white spherical).

To further validate the fermentation performance of F127-IEA-T15 in non-detoxified corn stover hydrolysate, we conducted continuous cell fermentation experiments to produce acid from the hydrolysate. The initial concentrations of glucose, FA, and vanillin in the corn stover hydrolysate were 5.079, 0.25, and 1.86 g/L, respectively. The hydrolysate was then concentrated to achieve a glucose concentration of 40 g/L, which served as the initial carbon source for subsequent F127-IEA-T15 cell-recycle fermentation experiments aimed at producing D-LA. The feasibility of using F127-IEA-T15 for D-LA production from corn stover hydrolysate was assessed (Figure 3). The results showed that the D-LA production rate in the first cycle was only 0.42 ± 0.04 g/L h, significantly lower than the production rate observed during glucose fermentation (Figure 2). This result suggests that the presence of 2 g/L FA and 14.88 g/L vanillin in the fermentation substrate had a substantial inhibitory effect on F127-IEA-T15. The immobilized cells required time to adapt to the new environment, with some T15 cells either perishing or nearing death under these conditions. However, with increased recycling cycles under identical fermentation conditions, there was a marked improvement in the production rate. Over 30 fermentation batches (30 days), the average D-LA production rate by F127-IEA-T15 cells from corn stover hydrolysate reached 1.29 ± 0.09 g/L/h, with the wear rate of F127-IEA remaining below 5%. During ten consecutive batches using corn stover hydrolysate as the sole carbon source, both the yield and efficiency of F127-IEA-T15 were comparable to those observed when glucose was used as the sole carbon source in previous experiments. The result is similar as other studies (Supplementary Table S1). These findings conclusively demonstrate that F127-IEA, as an immobilization material for *Lactobacillus bulgaricus* T15, facilitates effective cellulose utilization while mitigating the inhibitory effects of phenolic by-products generated during corn stover pretreatment.

### 3.4 The adsorption characteristics of F127-IEA

**Figure 4:**
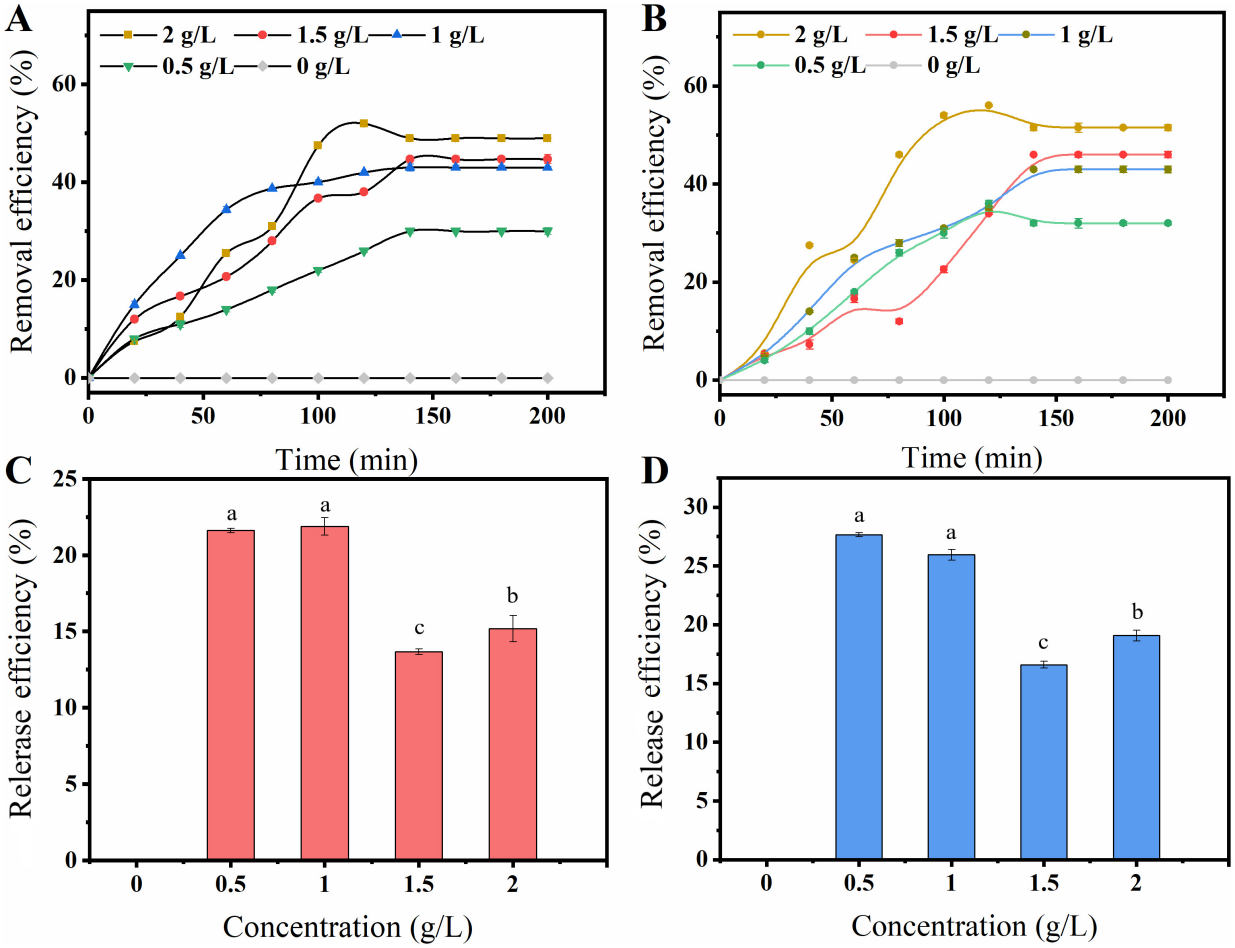
The adsorption and release property of F127-IEA on vanillin and FA. Take F127-IEA in vanillin solutions of 0, 0.5, 1, 1.5, and 2 g/L, and allow for adsorption to occur over a period of 0–200 min. After a specified adsorption time, measure the remaining concentration of vanillin or FA in the solution to characterize the adsorption capacity of F127-IEA towards vanillin **(A)** and FA **(B)**; After allowing F-127-IEA to fully adsorb in solutions of vanillin or FA at concentrations of 0, 0.5, 1, 1.5, and 2 g/L, the samples were maintained in an aqueous solution for 1 h. Subsequently, the concentrations of vanillin or FA in the solution were measured to characterize the release capacity of F127-IEA towards vanillin **(C)** and FA **(D)**. Different letters (a, b, c) indicate significant differences among groups (*P* < 0.05).

To further analyze how the production capacity of F127-IEA-T15 is protected in the presence of cytotoxic substances such as phenolics, and to determine whether the hydrogel adsorbs toxic and harmful substances, we conducted adsorption experiments with vanillin and FA. The ability of porous matrix-like materials to adsorb phenolics is crucial for enhancing the tolerance of immobilized cells to phenolic inhibitors (Du H. et al., 2020). The presence of small pore windows and closed surfaces in the material exhibits a molecular sieve effect, enabling us to investigate the correlation between such structures and selective phenol adsorption performance (Hu et al., 2016). It is speculated that the adsorption of phenols by F127-IEA involves both physical adsorption and chemical bonding. Moreover, the permeability coefficient of the porous matrix material hinders the transfer of inhibitors within it, thereby reducing their contact with cells inside (Sanada and Kimura, 1997). Millik et al. (2019) demonstrated, for the first time, the significant contribution of functional groups in materials, which play a decisive role in chemical bond adsorption. Furthermore, Wang et al. incorporated vermiculite with a surfactant (imino), resulting in a substantial increase in its adsorption capacity due to hydrogen bonds and π-π interactions with the adsorbent (FA, vanillin). Therefore, we speculate that F127-IEA can help *Lactobacillus bulgaricus* T15 maintain its cell viability and fermentation performance by adsorbing cytotoxic substances (FA and vanillin). This hypothesis was supported by the results shown in Figure 3. The adsorption capacity of the hydrogels for phenol compounds was assessed, and it was observed that the adsorption of F127-IEA exhibited a direct correlation with the initial concentration of the adsorbate under isothermal conditions in identical settings. Equilibrium adsorption was achieved within 2 h. The adsorption rate of F127-IEA was 50% at an initial concentration of 2 g/L, whereas it decreased to 25 and 30% at concentrations of 0.5 and 0.1 g/L, respectively (Figures 4A, B). Notably, the concentrations of FA and vanillin in the solution remained unchanged after a duration of 2 h (Figures 4C, D). The diffusion of phenolics into the solution was hindered by concentration differences; instead, they were adsorbed onto the material's surface. Compared to F-BUM prepared by Butelmann, the F127-IEA gel synthesized in this study exhibits a higher propensity for adsorption of aromatics after removal of the two methyl groups (Wang et al., 2021).

### 3.3 The phenolics tolerance of F127-IEA-T15

**Figure 5:**
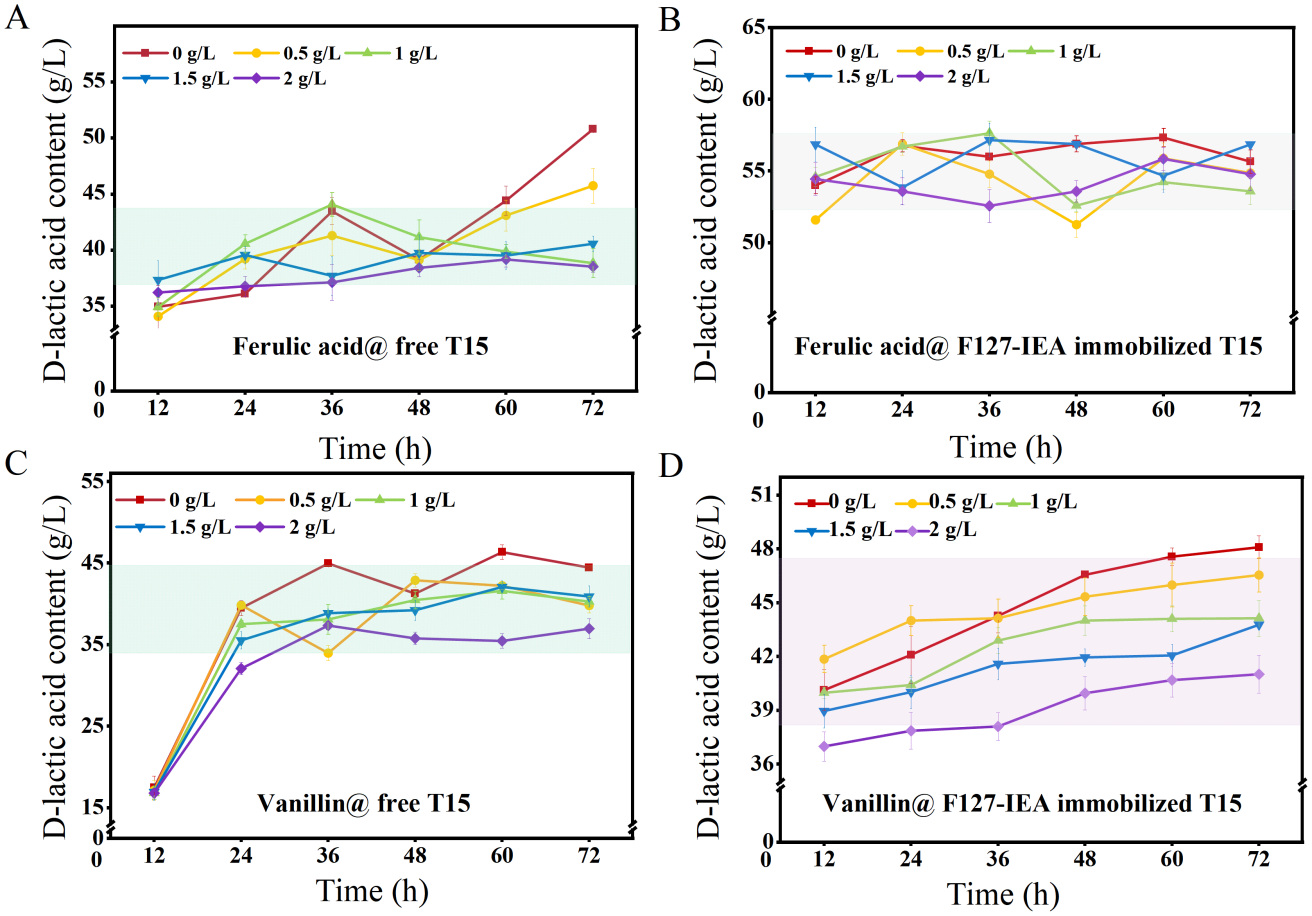
The tolerance property of F127-IEA-T15 to FA and vanillin. D-LA production with free T15 **(A)** and F127-IEA-T15 **(B)** at FA concentration of 0, 0.5, 1, 1.5, and 2 g/L; D-LA production with free T15 **(C)** and F127-IEA-T15 **(D)** at vanillin concentration of 0, 0.5, 1, 1.5, and 2 g/L.

The tolerance analysis of F127-IEA immobilized T15 cells to phenolic inhibitors was conducted, with D-LA yield serving as an indicator and free cells used as the control group (Figure 5). The results demonstrated that F127-IEA immobilized T15 cells exhibited enhanced stability at high FA concentrations, achieving a single batch D-LA production ranging from 50 to 58 g/L compared to 32–50 g/L for free bacteria (Figures 5A, B). In our study, we meticulously investigated the growth kinetics of free *Lactobacillus bulgaricus* T15, FA, and vanillin in relation to lactate and glucose production (Supplementary Figure S3). The findings revealed that *Lactobacillus bulgaricus* T15 exhibited distinct growth patterns in response to varying concentrations of FA and vanillin. Notably, these growth kinetic observations were corroborated by the production of D-LA by the free *Lactobacillus bulgaricus* T15, which was also modulated by the presence of FA and vanillin.

The adsorptive properties of F127-IEA substantially mitigated the inhibitory impact of ferulic acid and vanillin on T15 cell activity, an effect that was mirrored in the D-LA yield of both free and immobilized cells. As depicted in Figure 4, the adsorption efficacy of F127-IEA escalated with increasing concentrations of FA and vanillin in the solution. This heightened adsorption rate ensures that the cells remain unimpeded by inhibitory compounds throughout extended fermentation periods, thereby preserving the efficiency of D-LA production. In the presence of elevated vanillin concentrations, the fermentation process initiated earlier in immobilized T15 cells, with these bacteria entering the fermentation stage at 12 h, while free bacteria only commenced fermentation after 24 h of inoculation (Figure 5C). The immobilized T15 strain exhibited an overall single-batch D-LA production ranging from 40 to 48 g/L, whereas the free cells showed a production range of 35–45 g/L (Figure 5D). Importantly, the F127-IEA immobilized T15 cells demonstrated a higher D-LA yield in fermentation using non-detoxified corn stover compared to previous studies (Wang et al., 2021). The findings suggest that the adsorption of phenolics by F127-IEA at high concentrations had a lesser negative impact on the cell membrane of T15 cells. Additionally, the formation of a cell population through immobilized cell fermentation exhibited synergic effects, resulting in enhanced D-LA production.

### 3.4 Application and prospect of F127 hydrogel in different fields

F127 hydrogel biomaterial has attracted significant consideration in wound healing and tissue repair due to their outstanding properties, such as temperature sensitivity, injectability, biodegradability, and their ability to maintain a moist environment in medical applications. For example, F127-CHO hydrogels are commonly used in burn wound healing (Yang et al., 2020), F127-NH_2_ hydrogels have demonstrated excellent efficacy in preventing postoperative adhesions, and F127-DA hydrogels are more suitable for the healing non-sutured wounds (Du X. et al., 2020).

In our study, the F127-IEA hydrogel, a synthetic material, has been innovatively employed for the first time for microbial immobilization to enhance D-LA production. This hydrogel exhibits the non-toxicity and eco-friendliness characteristic of F127 hydrogels, making it a highly desirable material for bioprocess applications. Furthermore, its superior mechanical properties are instrumental in sustaining continuous microbial fermentation processes, ensuring the longevity and stability of the fermentation system. Future research endeavors could harness the potential of F127-IEA hydrogel immobilized cell technology for the bioproduction of energy-rich substances such as ethanol (Ramos et al., 2023), as well as rare saponins (Zhang et al., 2024) and terpenoids (Han and Miao, 2024). This approach has the promise to enhance production efficiency, bolster sustainability, and mitigate environmental impact, thereby contributing to a more eco-friendly and economically viable bioprocess industry.

## 4 Conclusion

In this study, we developed and characterized a novel hydrogel, F127-IEA, using SEM and EAS techniques. The Hencky stress, WHT, and embedding rate results confirmed the superior material properties of F127-IEA for cell immobilization. Additionally, F127-IEA's enhanced adsorption capacity and permeation coefficient significantly improved cell resistance to phenolic compounds, thereby reducing their cytotoxicity. This was further validated through adsorption rate tests and acid yield tolerance assays. This capability is especially crucial for fermentation processes that utilize biomass feedstocks, which frequently contain inhibitors that can impede microbial growth and metabolism. The distinctive mechanical attributes and biocompatible nature of the material under investigation render it a prospective candidate for enhancing cell viability in adverse environmental conditions, potentially making a significant contribution to the field of environmental bioremediation. Remarkably, F127-IEA maintained excellent D-LA yields even after 30 days of storage at −80°C or 150 days of recirculation in its immobilized form. When applied in non-detoxified corn stover fermentation with *Lactobacillus bulgaricus* T15 immobilized on the F127-IEA matrix, it exhibited exceptional fermentation performance, achieving D-LA yields 12–48% higher than those reported in recent studies. This work presents a novel strategy to enhance resource utilization and reduce biorefining costs by efficiently leveraging non-detoxified corn stover. These findings underscore the potential of F127-IEA as a robust and efficient material for cell immobilization and D-LA production, marking significant progress in biorefining and industrial fermentation processes.

## Data Availability

The raw data supporting the conclusions of this article will be made available by the authors, without undue reservation.
