# Supplementary material for: Enhancing D-lactic acid production from non-detoxified corn stover hydrolysate via innovative F127-IEA hydrogel-mediated immobilization of Lactobacillus bulgaricus T15
Source: Front Microbiol. 2024 Dec 5;15:1492127. doi: 10.3389/fmicb.2024.1492127 (PMC11655503; doi:10.3389/fmicb.2024.1492127)
Supplement: Supplementary file 1 [file Data_Sheet_1.docx]

**Caption of Figure**

**Figure s1**. Preparation, separation, and purification process of modified F127-IEA

**Figure s2**. Synthesis and characterization of F127-IEA. F127-IEA preparation by adding ethyl isocyanate containing imino groups to both ends of F127 polymer (A); high mechanical strength F127-IEA solidified according to the thermo-sensitive

**Figure S3** The adsorption property of free Lactobacillus bulgaricus T15 growth kinetic on FA (A) and vanillin (B)


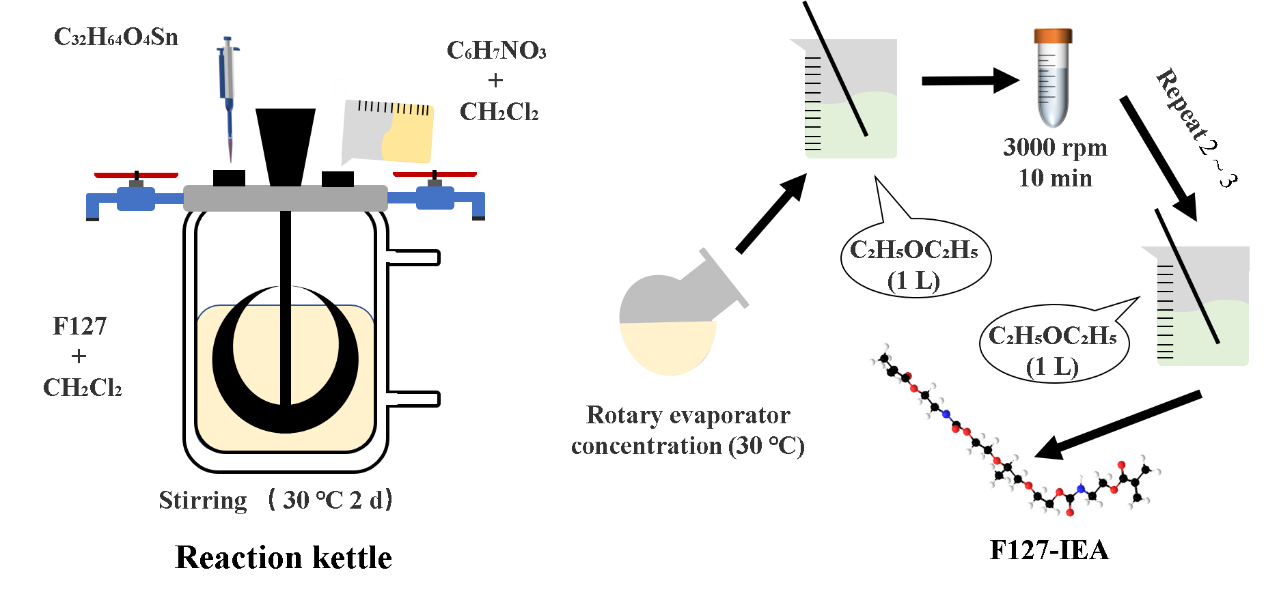


**Figure s1**. Preparation, separation, and purification process of modified F127-IEA.


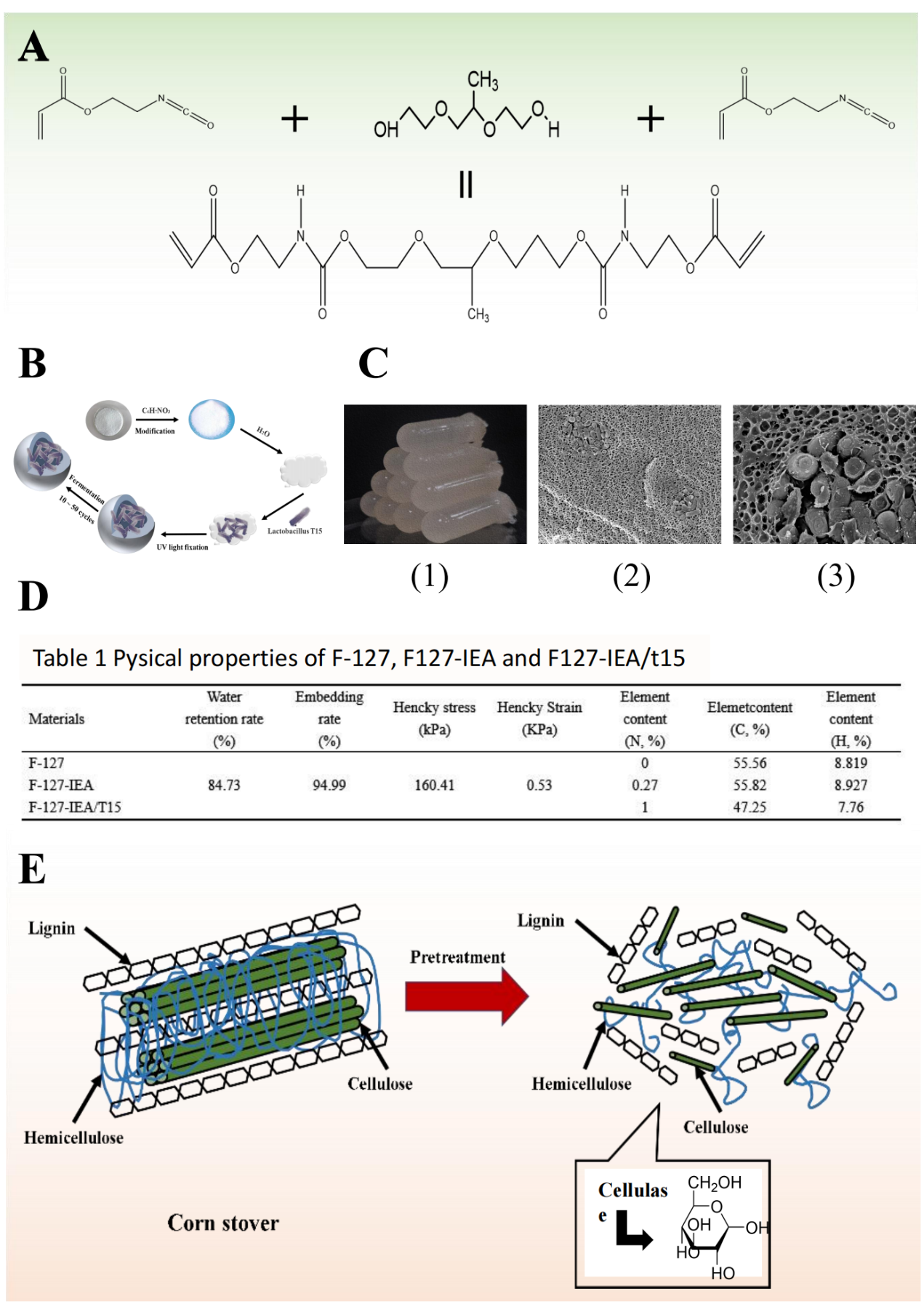
**Figure S2** Synthesis and characterization of F127-IEA. F127-IEA preparation by adding ethyl isocyanate containing imino groups to both ends of F127 polymer (A); high mechanical strength F127-IEA solidified according to the thermo-sensitive

**Caption of Tables**

**Table 1.** LA or D-LA yields from food or non-food resources using immobilization techniques.

**Table s1 production rate from food or non-food resources using immobilization techniques**

| Strain | Materials | Substrate | D-LA production rate (g/L h) | References |
| --- | --- | --- | --- | --- |
| *Lactobacillus rhamnosus* ATCC 53103 | Calcium alginate | Carob waste | 1.22 | [16] |
| *Rhizopus oryzae* PTCC 5263 | Calcium alginate | Soluble potato starch | 0.37 | [17] |
| *Lactobacillus rhamnosus* | mesoporous silica-based | Glucose | 0.74 | [18] |
| *Lactobacillus paracasei subsp* | polyurethane foam | Glucose | 1.60 | [19] |
| *Acid- ipropionici* ATCC 4875 | Fibrous-bed bioreactor | Glucose | 0.99 | [20] |
| *Lactobacillus rhamnosus* ATCC7469 | The BSG, solid remains after BSG and MR hydrolysis | Brewer’s spent grain | 1.15 | [21] |
| *Sporolactobacillus inulinus* Y2-8 | fibrous bed bioreactor | Corn flour | 1.62 | [22] |
| *Lactiplantibacillus pentosus* B329 | cheese whey | Bacteria | 0.91 | [23] |





**Figure S3** The adsorption property of free Lactobacillus bulgaricus T15 growth kinetic on FA (A) and vanillin (B)
